# Data on the effect of miR-15b on the expression of INSR in murine C2C12 myocytes

**DOI:** 10.1016/j.dib.2017.10.053

**Published:** 2017-11-01

**Authors:** Won-Mo Yang, Kyung-Ho Min, Se-Whan Park, Wan Lee

**Affiliations:** a Department of Biochemistry, Dongguk University College of Medicine, Gyeongju-si, Gyeongsangbuk-do 38067, Republic of Korea; b Endocrine Channelopathy, Channelopathy Research Center, Dongguk University College of Medicine, Goyang-si, Gyeonggi-do 10326, Republic of Korea

**Keywords:** MicroRNAs, miR-15b, Myocyte, INSR, IRS-1

## Abstract

The ectopic expression of miR-15b is linked causally to impaired insulin signaling in human HepG2 hepatocytes through the suppression of INSR (Yang et al., 2015) [1]. In this data article, we further examined the effect of miR-15b on insulin signaling in a murine skeletal muscle cells, C2C12 myocytes. Although the 3’UTR of mouse INSR mRNA has an appropriate binding site for miR-15b based on TargetScan analysis, the ectopic expression of miR-15b did not suppress the expression and insulin-stimulated phosphorylation of insulin signaling intermediates in C2C12 myocytes. A more detailed understanding of the effects of miR-15b on hepatic insulin resistance can be found in “Obesity-induced miR-15b is linked causally to the development of insulin resistance through the repression of the insulin receptor in hepatocytes” (Yang et al., 2015) [1].

**Specifications Table**

**Table:** 

Subject area	*Cell Biology, Biochemistry*
More specific subject area	*Obesity, MicroRNA, Metabolism*
Type of data	*Figures and text*
How data was acquired	*TargetScan analysis and immunoblotting*
Data format	*Analyzed*
Experimental factors	*Transfection of miR-15b, Treatment of insulin, Analysis of the expression and phosphorylation of insulin signaling intermediates*
Experimental features	*C2C12 myocytes were transfected with scRNA or miR-15b mimic. For insulin stimulation, 100 nM of insulin was treated during the last 30 min of incubation.*
Data source location	*Dongguk University School of Medicine, Gyeongju-si, Gyeongsangbuk-do 38067, Korea*
Data accessibility	*The data are available with this article*

**Value of the data**

• The data are useful for understanding the putative binding sites of miR-15b on the 3′UTR of human and mouse INSR mRNA.
• The effect of miR-15b on the insulin signaling pathway in mouse skeletal muscle cells.
• The data can be compared with the target of miR-15b between hepatocytes and myocytes.

## 1 Data

**Fig. 1:**
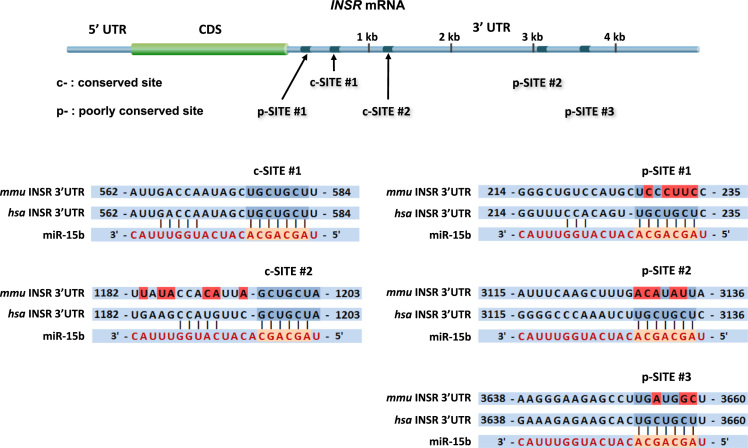
*Putative targeting sites of miR-15b in the 3′UTRs of murine and human INSR.* The miR-15b targeting INSR 3′UTR was analyzed using TargetScan. The seed sequence of miR-15b predicted to target *INSR* 3′UTRs (orange background) was identified in murine (*mmu*) and human (*has*).

**Fig. 2:**
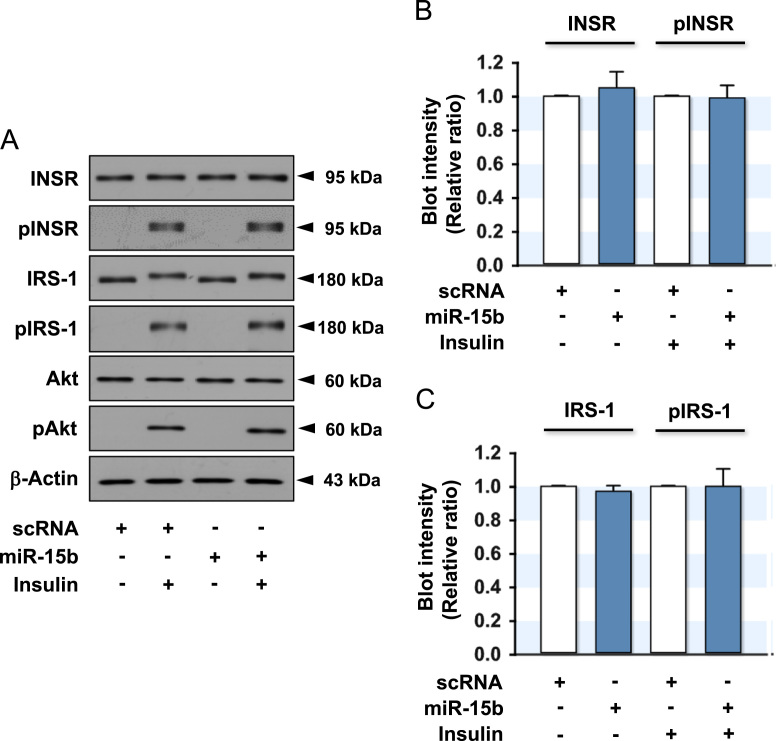
*Effect of miR-15b on the expression and phosphorylation of insulin signaling molecules.* C2C12 myocytes were transfected with the scRNA (200 nM) or miR-15b (200 nM) mimic. After 48 h transfection, the cells were incubated in the presence or absence of insulin (100 nM) for 30 min and subjected to immunoblotting. (A) Representative immunoblots obtained from C2C12 myocytes are shown in A. (B) The expression (INSR) and phosphorylation of INSR (pINSR) were normalized to the amount of β-actin. (C) The expression (IRS-1) and phosphorylation of IRS-1 (pIRS-1) were normalized to the amount of β-actin. The values are the relative ratio, where the intensity of the scRNA control was set to one, and expressed as the means ± SEM from three independent experiments.

Intake of high saturated fatty acid (SFA) in diets results in ectopic lipid accumulation in the liver and skeletal muscle, which is a major risk factor for insulin resistance, type 2 diabetes, and metabolic syndrome [2]. The dysregulation of certain miRNAs targeting the insulin signaling molecules is closely associated with diet-induced obesity, which participates actively in the pathogenesis of insulin resistance [3], [4]. In a previous report, the transfection of miR-15b suppressed the expression of INSR by targeting *INSR* 3′UTR directly, leading to impaired insulin signaling in human HepG2 hepatocytes [1]. This article provides accompanying data to analyze further the effect of miR-15b in the C2C12 cell line derived from murine skeletal muscle cells. Compared to the sequences on the 3’UTR of human INSR mRNA, which contains five different seed sequence binding sites for miR-15b, the 3′UTR of mouse *INSR* mRNA has only two seed binding sites for miR-15b according to TargetScan analysis (Fig. 1). Among those, the conserved site #1 is considered as an appropriate binding site for miR-15b in mice. C2C12 myocytes were transfected with the scRNA control or miR-15b mimic, as described in the Method section, and the expression and phosphorylation of insulin signaling intermediates, such as INSR, IRS-1 and Akt, were determined in the presence or absence of insulin stimulation (Fig. 2). In the murine muscle cells, in contrast to previous observations in human hepatocytes [1], the ectopic expression of miR-15b did not suppress the expression and insulin-stimulated phosphorylation of insulin signaling intermediates. This data is associated with a previous research article entitled “Obesity-induced miR-15b is linked causally to the development of insulin resistance through the repression of the insulin receptor in hepatocytes” [1].

## 2 Experimental design, materials and methods

### 2.1 Cells, culture condition, and insulin treatment

C2C12, a mouse myoblast cell line, was obtained from ATCC (CRL-1772). The C2C12 cells was harvested in DMEM supplemented with 10% FCS and 1% penicillin-streptomycin (Gibco) in an atmosphere containing 5% CO_2_ at 37 °C. The undifferentiated cells from passages 4 to 12 were used in subsequent experiments. For insulin stimulation, the cells were cultured in serum-free medium for the last 5 h of the experiment, which was followed by a treatment with insulin (100 nM) for the last 30 min.

### 2.2 Antibodies and reagents

The anti-IRS-1 antibody was purchased from Upstate Biotechnology (Lake Placid, NY, US), and the antibody against phospho-IRS-1 (Tyr632) and β-actin were supplied by Santa Cruz Biotechnology (Santa Cruz, CA, US). The antibodies against INSR, phospho-INSR (Tyr1361), Akt, phospho-Akt (Ser473) were obtained from Cell Signaling Technology (Danvers, MA, US). ECL Western Blotting Detection Reagents from GE Healthcare (Buckinghamshire, UK) were used to visualize the immunoblot. Unless indicated otherwise, all other chemicals and materials were purchased from Sigma.

### 2.3 Transfection of miRNA mimics

The miRNA mimics and scRNA were purchased from Genolution (Seoul, Korea). C2C12 cells were transfected with the 200 nM mimics of scrambled control miRNA (scRNA) or miR-15b mimics using G-fectin (Genolution) according to the manufacturer's instructions. After 48 h transfection, the expression and phosphorylation of insulin signaling molecules were analyzed by immunoblotting.

### 2.4 Cell lysis and immunoblotting

C2C12 cells were washed three times with ice-cold PBS and lysed using a lysis buffer (ice-cold PBS containing 1% Triton X-100, phosphatase inhibitor cocktail II, and 0.2 mM PMSF) by homogenization. The lysates were mixed with 2X Laemmli buffer, and heated for 10 min at 100 °C. Gel electrophoresis was carried out by SDS–PAGE on 10 or 8% resolving gels, transferred and immunoblotted with various antibodies. The intensities of the immunoblots were determined by densitometry using an Alpha Imager HP scanning system (Alpha Innotech, San Leandro, CA, US).

### 2.5 Database for *in silico* analysis

The miR-15b targeting *INSR* 3′UTR was analyzed computationally using publicly available algorithms (TargetScan: www.targetscan.org). The experimental values are expressed as the means ± SEM from three independent experiments. The significance of the difference was analyzed using a Student's *t*-test for unpaired data.
